# Rapid Plant Invasion in Distinct Climates Involves Different Sources of Phenotypic Variation

**DOI:** 10.1371/journal.pone.0055627

**Published:** 2013-01-30

**Authors:** Arnaud Monty, Jean-Philippe Bizoux, José Escarré, Grégory Mahy

**Affiliations:** 1 University of Liege, Gembloux Agro-Bio Tech, Biodiversity and Landscape Unit, Passage des Déportés, 2, B-5030 Gembloux, Belgium; 2 Centre d'Ecologie Fonctionnelle et Evolutive, UMR 5175, Route de Mende, 1919, B-5000 Montpellier, France; Swansea University, United Kingdom

## Abstract

When exotic species spread over novel environments, their phenotype will depend on a combination of different processes, including phenotypic plasticity (PP), local adaptation (LA), environmental maternal effects (EME) and genetic drift (GD). Few attempts have been made to simultaneously address the importance of those processes in plant invasion. The present study uses the well-documented invasion history of *Senecio inaequidens* (Asteraceae) in southern France, where it was introduced at a single wool-processing site. It gradually invaded the Mediterranean coast and the Pyrenean Mountains, which have noticeably different climates. We used seeds from Pyrenean and Mediterranean populations, as well as populations from the first introduction area, to explore the phenotypic variation related to climatic variation. A reciprocal sowing experiment was performed with gardens under Mediterranean and Pyrenean climates. We analyzed climatic phenotypic variation in germination, growth, reproduction, leaf physiology and survival. Genetic structure in the studied invasion area was characterized using AFLP. We found consistent genetic differentiation in growth traits but no home-site advantage, so weak support for LA to climate. In contrast, genetic differentiation showed a relationship with colonization history. PP in response to climate was observed for most traits, and it played an important role in leaf trait variation. EME mediated by seed mass influenced all but leaf traits in a Pyrenean climate. Heavier, earlier-germinating seeds produced larger individuals that produced more flower heads throughout the growing season. However, in the Mediterranean garden, seed mass only influenced the germination rate. The results show that phenotypic variation in response to climate depends on various ecological and evolutionary processes associated with geographical zone and life history traits. Seeing the relative importance of EME and GD, we argue that a “*local adaptation* vs. *phenotypic plasticity*” approach is therefore not sufficient to fully understand what shapes phenotypic variation and genetic architecture of invasive populations.

## Introduction

When spreading into a new range, plant invaders often experience variation in environmental conditions. Climate, for instance, often varies gradually over the invasion range at both regional and continental scales. Clinal variation in life history traits (i.e. gradual variation along an environmental gradient), associated with climate, have been documented in several invasive plants [1], [2], [3]. Often, these patterns of population differentiation have been interpreted as resulting from adaptation [4], [5]. However, several mechanisms, or *sources of phenotypic variation*, act and interact to allow the persistence of plant populations under variable climatic conditions within the invaded range.

Within a species distribution, climatic variability generates heterogeneous selection pressures. Local adaptation (LA) can be defined as the result of selection pressures exerted by local environmental conditions on a population's genetic pool. Genetic differentiation of invasive plant populations can be the result of selection combined with restricted gene flow [6], but locally adapted genotypes can be maintained by selection despite gene flow among populations [7]. LA to environmental conditions other than climate has been shown to play a role in successful plant invasion [8]. However, to date, little evidence of LA to climatic conditions in the invaded range has been shown [9], [10].

The genetic structure of alien species in the invaded range is not only the result of selection. Genetic drift (GD), i.e. the random fluctuation of allele frequencies, can also shape the genetic structure [11]. Keller and Taylor [12] illustrated that GD in invasive plants can be regulated by colonization history.

Phenotypic plasticity (PP) is the change in phenotypic expression of a genotype in response to environmental conditions [13]. Davidson et al. [14] showed that invasive species tend to show higher PP than co-occurring non-invasive species, indicating an important role of PP in invasiveness, but other studies have found different results [15]. Richards et al. [16] made an important distinction between PP in fitness traits (estimates of reproductive success) *vs* non-fitness traits and proposed that a successful invader can benefit from plasticity as i) a *Jack-of-all-trades*, able to maintain fitness across a range of environmental conditions, notably through the plasticity in morphological and physiological traits and/or ii) a *Master-of-some*, better able to increase fitness in favorable environmental conditions.

In the literature, PP and LA have often been studied as two potential mechanisms for the success of invaders (“local adaptation vs. phenotypic plasticity” approach [17], [18], [19]).

Environmental maternal effects (EME) can also modulate phenotypic variation in plant populations and mediate population success across generations. In a broad sense, EME are maternal phenotype influences on offspring phenotype that occur via mechanisms exclusive of any genetic information [20]. In plants, these factors are typically mediated by seed size or seed mass, i.e. by means of a positive relationship between seed size, the probability and speed of germination, and subsequent seedling size (reviewed in [21]).

These different sources of phenotypic variation, associated with climate, have been studied in invasive plant species [2], [10], [22], [23]. However, to our knowledge, studies have not been attempted to simultaneously assess LA, GD, PP and EME in response to climate in a quantitative framework. In contrast, potential sources of phenotypic variation have often been eliminated in order to study other sources in more detail, with the risk of overestimating the actual importance of the mechanisms in nature. Nevertheless, interactions between sources of phenotypic variation have been documented in plant species. For instance, since an individual's potential to develop a plastic response in life history traits (and notably seed provisioning) may have a genetic basis, PP and EME can be adaptive if promoted by natural selection [24]. Low genetic diversity, due to GD caused by repeated bottlenecks [25], can decrease a population's adaptive potential [26]. Also, evolution of particular traits through one mechanism can influence other sources of phenotypic variation. For example, genetic differentiation of seed dispersal traits was shown during the invasion history of *Senecio inaequidens* (Asteraceae) [27], and seed traits are known to be central in the role of EME [20], [21].

Reciprocal common gardens are useful tools to examine the mechanisms responsible for the phenotypic variation within plants [6], [10], [28]. Because they compare different origins of plants in their natural environments and the fitness of local and foreign populations can be directly compared, they can estimate the adaptive role of genetic differentiation (i.e. LA), PP and EME. LA is typically evidenced by increased fitness of local vs. foreign genotypes or populations within each test habitat [6], whereas PP is quantified by phenotypic differences between gardens at the genotypic or population levels [29]. Despite interest in these concepts, only a few reciprocal common garden experiments have been applied to invasive species [8], [9], [10].

In the present study, we explored phenotypic variation in response to climate and evaluated the extent of LA, GD, PP and EME in germination, growth, ecophysiological and reproductive traits at the population level. The first step of the present study was a reciprocal sowing experiment. An experimental garden was placed in each of the two most extreme climatic zones colonized by a rapidly invasive plant species after migrating from a single introduction site. We subsequently conducted a quantitative analysis of phenotypic variation in life history traits. The second step was a quantitative assessment of the species genetic structure using Amplified Fragment Length Polymorphism (AFLP) markers in order to better understand the patterns of genetic differentiation during invasion.

The model species *Senecio inaequidens* DC (Asteraceae) is considered a particularly suitable model for evolutionary studies [30], [31], [32], [33], [34], including a well-delimited time frame for population differentiation, i.e. approximately one century. The knowledge of a single introduction site, along with the well-documented invasion history, should provide a relatively fine-scale and non-ambiguous interpretation of the results.

## Materials and Methods

### Study species and invasion history

*S. inaequidens* is a herbaceous perennial native to Southern Africa, where it occurs as two co-existing cytotypes. Only tetraploids are reported in Europe [35]. Individuals can produce up to 1500 capitulae over a flowering period, with each capitulum bearing roughly one hundred achenes. Achenes (referred to as seeds) are mainly dispersed by wind [36]. Sexual reproduction is mainly allogamous and is initiated in late spring (May–June), approximately two months after massive germination, and continues through late autumn (November–December). *Ex situ* germination tests have indicated that germination is absent at temperatures below 2°C [37]. Entomophilous pollination with generalist pollinators is most common.

As a result of wool importation from South Africa to Europe during the late 19^th^ century, recorded independent introduction sites chronicle the species migration routes [38]. In southern France, the species was first recorded in the wool-processing centre of Mazamet in 1936 [38], [39], [40]. It was only after 1950 that the species began its expansion [40]. By the 1970s, the species had colonized areas of southwestern France, well within the first introduction region. By the early 1980s, the species reached the Mediterranean coast from Mazamet [40], where it experienced much drier and warmer climatic conditions [5]. Its expansion continued south towards the Eastern Pyrenean region, and was first recorded in Nohèdes (altitude ca. 800 m) in 1987 [41]. This uphill colonization continued [41], and the species is presently distributed at elevations reaching 1700 m, where the climate is much colder [5].

The species is not extensively propagated for ornamental purposes, thus reducing the risk of anthropogenic gene flow among regions. The particularly well-documented gradual expansion of *S. inaequidens* towards distinct climatic areas makes it an ideal model to study the response of invasive plant populations to climatic variation. A previous study indicated population differentiation along a climatic gradient in southwestern France [5]. However, the mechanisms responsible for genetic differentiation remain unknown.

### Seed populations

Three populations were selected in each of the following climatic zones: 1) the Mediterranean coast (*Armissan*, *Narbonne* and *Narbonne-Plage*); 2) the original introduction area, i.e. in the vicinity of the wool-processing industries of Mazamet (*Castaunouze*, *Mazamet* and *Hautpoul*); and 3) high plateaus in the Pyrenean region (*Egat*, *Enveitg* and *La Llagone*) (see Table S1). The three regions differ distinctly in climatic conditions. The Mediterranean coast experiences a typical Mediterranean climate with high summer temperatures and drought, while the Pyrenean plateau temperatures are much lower and rainfall occurs throughout the year (see Table S2). The original introduction area is characterized by moderate temperatures and the absence of summer drought [5]. The distances between populations within each climatic zone ranged from 1 to 20 km. Each population included at least 100 individuals, and was located along roadsides on rocky and/or gravel soils. In November 2007, fifteen plants were randomly selected per population and one ripe capitulum was collected from each plant for the reciprocal sowing experiment. In November 2008, seeds from 20 randomly selected individuals from each of nine populations were collected for AFLP analysis (see below).

### Reciprocal sowing experiment

Two identical random block common gardens were established in two of the climatic zones. One was representative of the Mediterranean climate (*Mediterranean garden*; alt.: 55 m; 43°38′N; 3°52′E), and the other under the Pyrenean mountain climate (*Pyrenean garden*; alt.: 1510 m; 42°30′N; 2°07′E). Table S2 shows the climatic conditions in the two gardens during the experiment, based on meteorological stations near the gardens. No garden could be installed in the original introduction area, but seeds from this climatic zone were used so that in each garden, three climatic zones (the two extremes and one intermediary) were considered.

We randomly selected two lots of 10 seeds from each collected capitulum. Seeds without obvious anomalies were chosen and weighed (Mettler Toledo analytical balance AG204, USA) to the nearest 0.1 mg (*seed mass*, SM). Each of the two lots was randomly assigned to a garden, with offspring from a field-collected plant representing a set of half or full siblings. Gardens were composed of 15 blocks of nine pots, and were surrounded by an additional row of potted plants to prevent edge effects. The pots were placed 80 cm apart to prevent competition and contained 2.5 l sand, 2.5 l compost and 0.5 l hydro-granulates. We used pots filled with a uniform substrate in order to eliminate environmental variation other than climate. On March 20^th^ (*Mediterranean garden*) and March 21^st^, 2008 (*Pyrenean garden*), the 10 seeds per lot were collectively sown in the pots. Pots were saturated with water and covered with protective light-permeable canvas (Plantex ProtecMax, DuPont, Belgium) until germination was complete to prevent seed predation and/or wind dispersal. Seedlings were counted every 2–3 days. The first emerged seedling in each pot was marked for measurement (totaling 135 per garden). Other seedlings were removed after counting. The *time to germination* was recorded, and represented the number of days between sowing and the emergence of the first seedling. The *germination rate* (%) was defined as the proportion of germinated seeds among the ten sown in each pot. During each month of the flowering period, all ripe flower heads were counted and clipped. For the last measurement, flowering capitulae and buds were counted as well as ripe flower heads. *Flower head production* was the sum of all capitulae over the growing season (or over the lifespan of the plant in the case of mortality), and was used as a proxy for fitness. In mid-October 2008, maximum height (h) and two perpendicular diameters (d1 and d2) were measured for all (surviving) plants. *Plant volume* (dm^3^), another fitness-related trait, was calculated as plant volume = π*h*(d1+d2)^2^/48 (volume of a cone). From September 22^nd^ to 26^th^, six leaves per plant were randomly collected in 10 of the 15 blocks in each garden. Selected leaves were fully expanded and did not show herbivore damage. Individual mean *specific leaf area* (SLA, m^2^.kg^−1^) and *leaf dry matter content* (LDMC, mg.g^−1^) were estimated according to Garnier et al. [42]. In late spring 2009, survival of the 270 plants was recorded. Plants were considered alive when shoots sprouted.

### Ethical statement

Since field populations were not on privately-owned or protected locations and no endangered or protected species were involved, specific permits were not required for seed collection. All necessary permits were obtained from the French *Office National des Forêts* (ONF) for the reciprocal sowing experiment.

### Statistical analysis of phenotypic traits

The mass of field-collected seeds was analyzed by applying a two-way ANOVA with climatic zone (Mediterranean, introduction, and Pyrenean) as the fixed factor and population (nested within climatic zone) as the random factor. In the case of a significant climatic zone effect, Tukey's multiple comparisons test was used.

Phenotypic traits in the reciprocal sowing experiment were analyzed. First, a four-way ANCOVA model was run with garden (G) and climatic zone (Z) as the fixed factors, population (P, nested within climatic zone) and block (B, nested within G) as the random factors and seed mass (SM) as the covariate. All possible interactions between factors were included in the model. The block factor and interactions were not significant, and were therefore removed from the model to increase the statistical power. A three-way ANCOVA model was subsequently used to analyze the effects of the interactions between the covariate and the climatic zone, garden and population factors. The interactions between factors and the covariate were then removed from the model to analyze the effects of individual factors, the covariate and interactions between factors. A correct test cannot be made for factors when covariate×factor interactions are included in the ANCOVA model, because the null hypothesis is an equal ordinate at the origin [43]. Due to strong interactions between the garden, other factors and the covariate, we analyzed the data from each garden separately (Table 1). We first ran a two-way ANCOVA to test these interactions (covariate: seed mass) with the climatic zone (fixed) factor and population (random, nested within climatic zone), including the interactions between the covariate and the factors. Again, the interactions between the covariate and the factors were removed from the model to adequately test the effects of the factors and the covariate.

**Table 1:** ANCOVA results.

	Time to germination			Germination rate			Plant volume			Flower head production			Specific leaf area			Leaf dry matter content		
All data	d.f.	F	P	d.f.	F	P	d.f.	F	P	d.f.	F	P	d.f.	F	P	d.f.	F	P
SM	1, 245	11.3	**0.001**	1, 251	17	**<0.001**	1, 233	10.1	**0.002**	1, 251	10.4	**0.001**	1, 157	0.09	0.766	1, 157	2.18	0.142
G	1, 245	2 047.50	**<0.001**	1, 251	370.1	**<0.001**	1, 233	15.2	**0.008**	1, 251	1	0.36	1, 157	178.73	**<0.001**	1, 157	277.8	**<0.001**
Z	2, 6	0.2	0.847	2, 6	1.7	0.252	2, 6	12.5	**0.005**	2, 6	0.9	0.447	2, 6	6.95	**0.017**	2, 6	2.78	0.128
P(Z)	6, 6	1.4	0.355	6, 6	4.2	**0.045**	6, 6	1.1	0.435	6, 6	1.1	0.444	6, 6	0.87	0.565	6, 6	1.69	0.267
GxZ	2, 6	1.3	0.348	2, 6	6.5	**0.032**	2, 6	0.4	0.696	2, 6	0.1	0.88	2, 6	5.5	**0.044**	2, 6	0.73	0.52
GxP(Z)	6, 236	0.8	0.555	2, 251	0.3	0.95	6, 233	1.3	0.496	2, 251	1.5	0.167	6, 157	0.65	0.688	6, 157	0.7	0.654
GxSM	1, 236	11	**0.001**	1, 242	0	0.883	1, 224	11.8	**0.001**	1, 242	6.7	**0.01**	1, 148	0.46	0.497	1, 148	0.52	0.471
ZxSM	2, 236	2.1	0.124	2, 242	0.6	0.561	2, 224	2.4	0.09	2, 242	2.8	0.062	2, 148	0.19	0.828	2, 148	0.81	0.445
PxSM(Z)	6, 236	1	0.46	6, 242	1.3	0.257	6, 224	0.5	0.78	6, 242	0.9	0.498	6, 148	0.42	0.864	6, 148	0.8	0.569
Mediterranean garden																		
SM	-	-	-	1, 125	10.42	**0.002**	1, 120	0.03	0.859	1, 125	0.39	0.531	1, 77	0.17	0.68	1, 77	0.18	0.669
Z	-	-	-	2, 6	0.04	0.6	2, 6	9.88	**0.01**	2, 6	0.22	0.806	2, 6	8.48	**0.012**	2, 6	9.27	**0.006**
P(Z)	-	-	-	6, 125	0.68	0.215	6, 120	2.08	**0.06**	6, 125	2.55	**0.023**	6, 77	0.66	0.683	6, 77	0.26	0.955
ZxSM	-	-	-	2, 117	0.81	0.448	2, 112	1.69	0.189	2, 117	7.67	**0.001**	2, 69	0.24	0.786	2, 69	0.03	0.973
PxSM(Z)	-	-	-	6, 117	1.25	0.288	6, 112	0.86	0.529	6, 117	1.6	0.155	6, 69	0.47	0.828	6, 69	0.7	0.654
Pyrenean garden																		
SM	1, 120	11.26	**0.001**	1, 125	6.98	**0.009**	1, 112	12.75	**0.001**	1, 125	11.35	**0.003**	1, 79	0.41	0.526	1, 79	2.71	0.103
Z	2, 6	0.23	0.801	2, 6	4.32	0.053	2, 6	4.87	**0.046**	2, 6	1.33	0.324	2, 6	3.6	0.073	2, 6	0.54	0.603
P(Z)	6, 120	1.22	0.303	6, 125	0.76	0.604	6, 112	0.99	0.433	6, 125	1.36	0.238	6, 79	0.67	0.677	6, 79	1.7	0.133
ZxSM	2, 112	2.01	0.139	2, 117	0.99	0.374	2, 104	1.23	0.296	2, 117	0.23	0.796	2, 71	0.67	0.517	2, 71	1.83	0.169
PxSM(Z)	6, 112	0.92	0.485	6, 117	0.86	0.527	6, 104	0.6	0.728	6, 117	1.01	0.425	6, 71	0.57	0.756	6, 71	0.69	0.662

Analysis of traits measured in the reciprocal sowing experiment. SM: seed mass (covariate); G: garden (fixed); Z: climatic zone (fixed); and P: population (random). The interactions between factors and the covariate (GxSM; ZxSM; and PxSM(Z)) were first tested in the ANCOVA model. These interactions were then removed from the model to test for factor effects, interactions between factors, and the covariate. As germination in all pots occurred between two rounds of observation in the Mediterranean garden, time to germination could not be analyzed for this garden (no variance). Significant results are in bold.

Each factor, covariate or interaction represents a source of phenotypic variation in relation to climate that potentially explains the variance in phenotypic traits. The garden factor reflects PP induced by climate at the population level. The climatic zone factor reflects genetic differentiation between distinct invaded regions, potentially due to LA if local populations outperform distant localities at the home site. If this is not observed, differences between zones are attributable to GD and colonization history. It has to be noted that LA can only be evidenced for populations from the Pyrenean and Mediterranean zones, since there was no garden in the original introduction area.

The random factor population corresponds to genetic differentiation within climatic zones. A significant garden×climatic zone interaction or garden×population interaction demonstrates genetic differentiation among and within climatic zones, respectively, on the level of PP in response to climate. The seed mass covariate suggests a role of EME on phenotypic variation. A significant seed mass influence indicates that seed provisioning by maternal plants affects offspring life history traits. A significant interaction with the garden factor demonstrates that the expression of EME is dependent on climate, whereas a significant interaction with the climatic zone or population factors indicates genetic control of EME. No transformation was necessary to meet the assumptions of statistical analyses. Analyses were performed with Minitab ver. 15.1.30 (Minitab Inc., State College, PA, USA). In order to analyze the effects of seed mass, garden, climatic zone, population and the interactions garden×climatic zone and garden×population on survival, we fitted a generalized linear model (GLM, R 2.8.0) to our data with a binomial error distribution and a logit link function. The predictors were added sequentially. Chi-square statistics were used to calculate the predictor significance.

### AFLP analysis

Seeds from 20 individuals from each population were sown under greenhouse conditions and one descendant per sampled individual was maintained. Descendants (totaling 180) were sampled for genetic analysis. Genomic DNA was extracted from 100 mg of fresh leaves using the DNeasy™ extraction kit (Qiagen, Germany). AFLP was performed as previously described [44] with minor modifications. Total genomic DNA (approximately 50 ng of DNA) was digested with 5 units of *Eco*RI and 2 units of *Mse*I (New England Biolabs, USA) and ligated to the appropriate double-strand adaptor (Eurogentec, Belgium) using 1 unit of T4-DNA ligase (Roche, Switzerland) in a total volume of 40 µl. Following two hours of incubation at 37°C, preselective amplification was performed with *Eco*RI-A and *Mse*I-C preamplification primers (Eurogentec, Belgium) from 3 µl of the digested-ligated DNA mixture (10× diluted). Amplifications were performed with a PTC 200 (MJ Research, USA) as follows: 2 min at 72°C; followed by 25 cycles of 30 s at 94°C, 30 s at 56°C, 2 min at 72°C; and a final extension step for 10 min at 72°C. Samples were cooled to 4°C. Preselective DNA amplification products were diluted 20×, and 5 µl were used in a selective PCR amplification. *Eco*RI primers were labeled with the 6-FAM fluorescent dye set. Selective PCR parameters were as follows: 10 min at 94°C; followed by 13 cycles of 30 s at 94°C, 1 min at 65–55.9°C (delta T = −0.7°C/cycle), 1 min at 72°C; followed by 23 cycles of 30 s at 94°C, 1 min at 56°C, 1 min at 72°C; and a final extension step for 10 min at 72°C. Samples were cooled to 4°C. A total of two primer pair combinations (*Eco*RI-AAC/*Mse*I-CAC and *Eco*RI-AAC/*Mse*I-CTC, Eurogentec, Belgium) were selected for twelve primer combinations on the basis of the quality and quantity of generated bands. Two repetitions of AFLP reactions were performed (separate extractions of the same individuals) on twenty individuals to test the repeatability of reactions. A blank control (i.e. no template) was included to detect the presence of contamination. Genotyping was conducted on an ABI PRISM 3100, using a pooled mix of 1.5 µl PCR product, 10 µl deionized formamide, and 0.2 µl GS500ROX size standard (Applied Biosystems, USA). AFLP patterns were visualized with GeneMapper software ver. 3.7 (Applied Biosystems, USA). Highly repeatable and reliably detectable loci were identified using duplicate samples and manual inspection before automatic scoring for the presence/absence (1/0) of selected bands in each individual. Bands of identical size amplified with the same primer were considered homologous.

Genetic structure was assessed by applying several approaches. The presence of differentiated gene pools in the overall sample without *a priori* geographic groupings was explored using the Bayesian clustering algorithm implemented in STRUCTURE ver. 2.1 [45]. Analyses were performed under the admixture model. Ten independent runs were carried out for each K value (number of clusters assumed) between 1 and 9, with parameters and model likelihood estimated at over 50,000 MCMC iterations, following a burn-in period of 20,000 steps. The maximum log-likelihood data value (L(K)), the minimum (L(K)) standard deviation associated with each K and the ΔK statistic value were analyzed to identify the number of clusters that best described the data. The mean percentage membership (qmean) from each individual to each K genetic cluster based on the ten best runs from 25 independent runs was assessed. An Analysis of Molecular Variance (AMOVA) was also performed to partition the genetic variance into three hierarchical levels: (1) within populations; (2) among populations; and (3) among climatic zones, using the software Arlequin ver. 3.01 [46]. Significance was tested on the basis of 10,000 random permutations. The percentage of polymorphic loci (p), the average expected heterozygosity (H) for each population/climatic zone and the overall genetic differentiation among populations (Fst) were obtained using AFLP-SURV software [47] and the Bayesian method with non-uniform prior distribution of allele frequencies, assuming Hardy-Weinberg equilibrium. The approach of Lynch and Milligan [48] was employed where the confidence interval for Fst and H was obtained by 5,000 permutation tests over all loci. Fst values between all populations and between climatic zones were estimated using the AFLP-SURV software, and their significance was determined using 5,000 permutation tests. A neighbor-joining unrooted dendrogram was constructed using Phylip ver. 3.69 [49] and Treeview ver.1.0 [50] to visualize genetic distances between all pairs of populations (Fst). Isolation by distance, the relationship between pairwise Fst values, and pairwise geographical and road distances were evaluated with a Mantel test using Arlequin ver. 3.01. Geographical distances were derived from maps and road distances were derived from www.viamichelin.fr. Road distance is potentially more relevant than geographic distance because *S. inaequidens* is known to follow roads and railways during colonization [27], [38]. Finally, the rarity index or DW (frequency-downweighted marker values) was estimated using the AFLPdat package in R [51]. DW was first applied by Schönswetter and Tribsch [52] for AFLP data, but is equivalent to range-downweighted values for species in historical biogeographical research. The presence of private and rare bands is characteristic of populations with a long *in situ* history, while a loss can be interpreted as a sign of GD. DW statistics were compared between climatic zones, based on population values using Mann-Whitney U tests.

## Results

### Field seed mass variation

Seed mass (i.e. mass of lots of 10 seeds) significantly differed between climatic zones (n  =  270; F_2,6_ = 9.26; *P* = 0.015) and between populations within zones (n  =  270; F_6,261_ =  4.15; *P* = 0.001). Mean ± SE were 37.6±0.56 10^−4^ g in the Pyrenean zone; 31.8±0.56 10^−4^ g in the introduction zone and 32.5±0.46 10^−4^ g in the Mediterranean zone. Tukey simultaneous tests indicated that seeds from the Pyrenean zone were significantly heavier than seeds from the introduction zone (T = −8.03; *P*<0.001) and the Mediterranean zone (T = 7.08; *P*<0.001). However, seed mass was not significantly different in the latter two zones (T = −0.952; *P* = 0.608).

### Reciprocal sowing experiment

The Mediterranean and Pyrenean gardens showed germination in 135 and 130 pots, respectively, where two and twelve plants did not bloom, and five and eight plants died before plant volume was measured. As germination in all pots occurred between two rounds of observation (between April 11^th^ and 14^th^), no variation in time to germination was recorded in the Mediterranean garden. However, in the Pyrenean garden, the germination window reached 60 days. The analysis of different sources of variation in phenotypic traits is presented in Table 1. A significant garden effect was detected for most traits, with the exception of flower head production (Table 1). Germination occurred on average 27.4 days later and at a lower rate (45.8% vs. 71.9%) in the Pyrenean garden than the Mediterranean garden. SLA and plant volume were higher in the Pyrenean garden; however, LDMC was lower.

**Figure 1:**
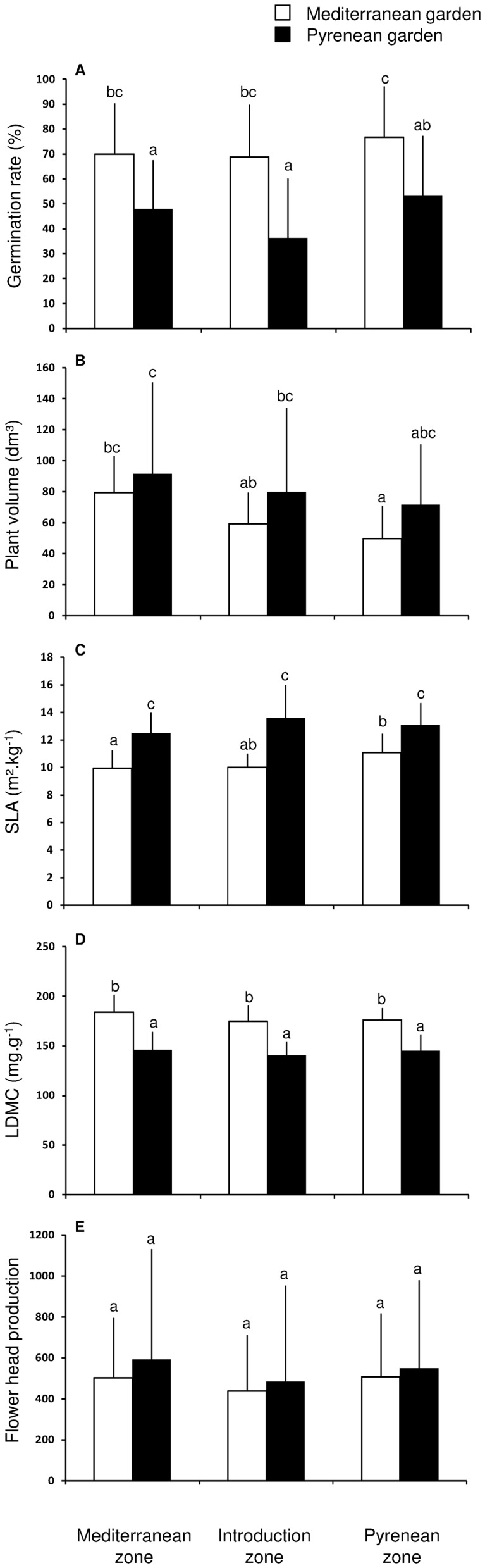
Trait variation (mean and standard deviation) in the reciprocal sowing experiment based on the climatic zone of origin and the experimental garden. Means with the same letter code are not significantly different (P < 0.05) based on the Tukey post hoc test.

Genetic differentiation among climatic zones was observed in plant volume, independent of the garden. Both in the Pyrenean and Mediterranean gardens, plants of Mediterranean origin grew bigger than other origins. In contrast, SLA and LDMC differences among zones were only significant under the Mediterranean climate (Table 1; Fig. 1).

Seed mass influenced all but leaf traits (SLA and LDMC). Exclusive of germination rate, the influence of seed mass was strongly conditioned by climate, indicated by a high garden×seed mass interaction. Seed mass strongly affected germination rate and timing, growth and reproductive output under the Pyrenean climate. However, it only influenced germination rate under the milder Mediterranean climate (Table 1).

Winter survival differed according to the garden (n  =  270; change in deviance  =  40.24; *P*<0.001), with 57 and 13 plants surviving in the Mediterranean and Pyrenean gardens, respectively. The predictor population was also significant (n  =  270; change in deviance  =  14.34; *P* = 0.046). Other predictors did not affect survival (n  =  270; change in deviance ranging from 0.07 to 6.63; all *P*>0.05).

### AFLP analysis

A total of 106 scorable fragments were obtained, with 94% polymorphism observed. Differentiation among populations was low but significant (Fst  =  0.0161, *P*<0.001). Bayesian analysis in STRUCTURE was applied for the overall analysis of individuals, which yielded the best clustering of data for K = 1, ((LnP(D|K = 1)) = −6401), suggesting the sample is comprised of a single genetic unit.

**Figure 2:**
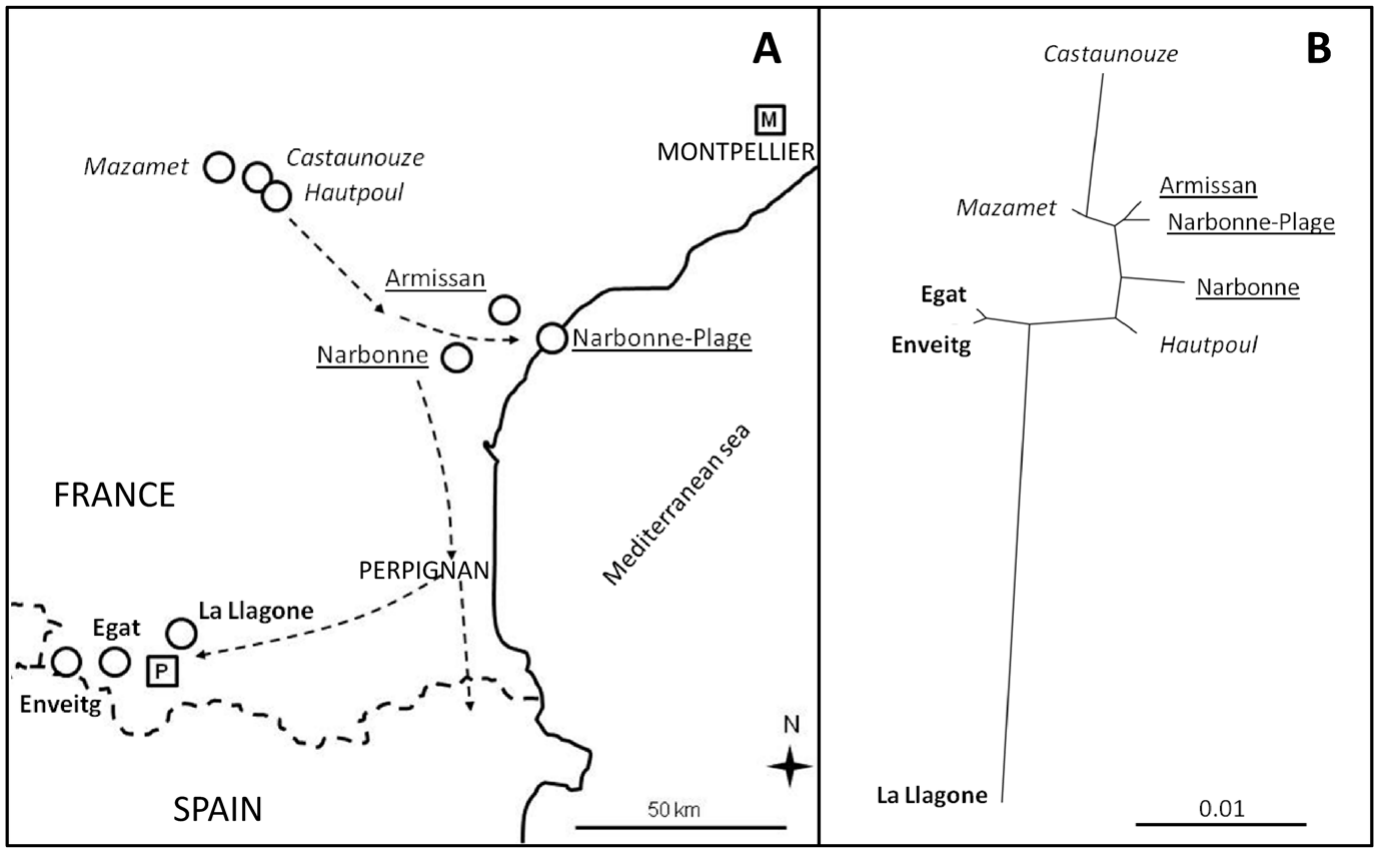
The role of invasion history in shaping the genetic structure of *S. inaequidens* in southern France. (A) Sampled *Senecio inaequidens* populations in the primary introduction area (italics); the Mediterranean zone (underlined); and the Pyrenean zone (bold), along with the progressive expansion chronology of the species in southern France derived from Guillerm et al. [40] and Monty and Mahy [27]. Squares represent experimental gardens (M: Mediterranean garden; P: Pyrenean garden). Countries and cities in capital letters facilitate the orientation of the reader. (B) Neighbor-joining unrooted dendrogram based on genetic distance between all pairs of populations (Fst).

One percent of the total molecular variance was due to differentiation among climatic zones (*P* = 0.031), 2.9% to differentiation among populations within climatic zones (*P*<0.001) and 96.1% to within-population variation. The Pyrenean zone was primarily responsible for climatic zone differences, exhibiting significant Fst associated with the Mediterranean zone (Fst  =  0.0178, *P*<0.001) and the introduction zone (Fst  =  0.0142, *P*<0.001). The pattern of genetic structure also showed high genetic differentiation between populations within climatic zones, with the exception of the Mediterranean zone. Pairwise genetic differentiation (Fst) between populations ranged from 0 to 0.051. Fst values were significant (P < 0.001) between the *La Llagone* population and all other populations (mean ± SD  =  0.039±0.007), and significant (*P*<0.05) between *Enveitg* and *Mazamet*; *Egat* and *Armissan*; and between *Castaunouze* and *Egat*, *Hautpoul* and *Narbonne*. This pattern was confirmed by the neighbor-joining unrooted dendrogram (Fig. 2), which clearly shows *La Llagone* was the most differentiated population. The Mediterranean populations formed a rather homogeneous group with origins in the genetic pool from the introduction site. The Pyrenean populations comprised a heterogeneous group more distant from the other populations. The Cerdagne plateau (*Enveitg* and *Egat*, Fig. 2) populations were more closely allied, while the *La Llagone* population was distinct.

The pairwise Fst values among the nine populations were correlated with pairwise geographic and road distances (Mantel test: r  =  0.32, *P*  =  0.017; and r  =  0.41, *P*  =  0.006 for geographic and road distance, respectively). The species level global genetic diversity (HT) was 0.2964 (SE  =  0.004814). Genetic diversity (H) of individual populations ranged from 0.267 (*Castaunouze*) to 0.316 (*La Llagone*) (mean ± SE  =  0.2917±0.002143) (Table S1). Differences in genetic diversity were not observed between climatic zones (Table S1). DW statistics ranged from 12.48 (*Enveitg*) to 16.2 (*Narbonne-Plage*) (mean ± SE  =  14.1±1.31). Pyrenean populations (mean DW  =  12.89) presented globally lower DW values than other populations (Mann-Whitney U-test, *P* = 0.029).

## Discussion

In the present study, we analyzed the phenotypic variation induced by climatic variation within the invaded range of *S. inaequidens*. Our approach voluntarily eliminated phenotypic variation related to other (e.g. edaphic or biotic) environmental factors to better examine the role primary sources of phenotypic variation play in the rapid and successful invasion of an introduced species in distinct climates. Our analysis of LA, PP, EME and GD therefore only corresponds to how the species responded to climatic variation during its invasion in southern France. To encompass climatic variation and genetic variability in this invaded range, we considered populations from three distinct climatic zones: the original introduction area (Mazamet) and two areas situated in the most extreme climates in this region, the high Pyrenean mountains and the Mediterranean coast. Since we could not set up an experimental garden in Mazamet, our analysis of LA, PP, EME and GD is exhaustive only for populations from the Pyrenean and Mediterranean regions. Populations from the original introduction area must essentially be seen as representative of an intermediary climate to understand the patterns of variation in the considered invaded range.

The results of the reciprocal sowing experiment and genetic analyses revealed that phenotypic variation in relation to climate involved more sources of phenotypic variation than previously reported in this species. Clinal differentiation in growth traits, as previously observed [5], was verified in the present study, but little evidence supported the pattern as adaptive. Furthermore, data suggested neutral genetic differentiation during species expansion, presumably due to GD. PP influenced many traits, but its influence on fitness proxies was low. EME mediated by seed mass were a major source of fitness and phenotypic variation under Pyrenean climatic conditions, whereas seed mass only affected germination rates along the Mediterranean coast.

### Phenotypic plasticity in relationship to climate

PP is more directly measured by comparing phenotypes of the same genotype in different environments. However, estimates of PP at the population level allow for a more realistic evaluation of its ecological role in natural populations [16], [53]. This approach enabled us to see a clear difference between plants growing under Pyrenean and Mediterranean climates for most traits, indicating PP in response to climate in germination, development and ecophysiology in *S. inaequidens*. However, over the growing season, reproductive output did not differ between gardens. Therefore, *S. inaequidens* appears capable of successful reproduction throughout its invasion range in southern France, despite different plastic responses. This corresponds to the *Jack-of-all-trades* situation described by Richards et al. [16], where through high PP in non-fitness traits (i.e. germination, growth and leaf physiology), the invader maintains fitness in different environments. In the Pyrenean zone, delayed germination was balanced by continuous growth, and in the Mediterranean zone, earlier spring germination was followed by summer drought, inhibiting plant development (*data not shown*). SLA and LDMC reflect a fundamental trade-off in plant function between a rapid production of biomass (high SLA, low LDMC) and increased drought resistance (low SLA, high LDMC). These traits are well-known to respond to environmental conditions [54]. In our study, PP influenced these traits. Individuals likely developed reduced SLA and increased LDMC under drought conditions in the Mediterranean relative to Pyrenean conditions.

The significant interaction between climatic zone and garden indicated that plants of Pyrenean origin were less plastic in SLA than others. This suggests that lower PP did not counteract the colonization of remote areas.

Similar reproductive output in both gardens indicated similar average one-year fitness in both climates. However, due to increased mortality following the harsher Pyrenean winter, longer-term fitness should be higher in the Mediterranean, enhancing future invasion.

### Genetic differentiation: local adaptation, genetic drift and invasion history

Since plant size can be considered a proxy for fitness [55], and size reduction is a common feature of mountain populations [56], clinal trends in growth traits along altitudinal transects in *S. inaequidens* are interpreted as a sign of LA [5]. However, the present study does not support this hypothesis. In the case of LA, a “home-site” advantage should be demonstrated in each garden. However, plants originating in warmer climates grew consistently larger regardless of the garden (Fig. 1). In addition, the variation in reproductive output and survival did not support an LA pattern, i.e. a home-site advantage. Growth trait clinal variation observed in *S. inaequidens* under the two climates could instead be the result of genetic differentiation in allocation pattern, with plants originating from the Mediterranean zone allocating more resources to growth than reproduction and Pyrenean plants allocating more resources to reproduction, e.g. through increased seed mass.

Since our approach specifically focused on phenotypic variation in response to climate, the lack of evidence for LA in the present study does not exclude that populations may have adapted to other environmental parameters. A recent study [57] suggested that the local-scale competitive regime acts as an important selection pressure within the invaded range of *S. inaequidens*. Further research is needed to fully understand the clinal trend in plant size in this species, but the present study suggests climate does not act as a major selection pressure.

In contrast, evidence for neutral genetic differentiation was detected within the invaded area in southern France. Despite a limited number of loci, the AFLP analysis helped to better understand the role of GD and colonization history in the genetic structure of the species. Differentiation was low, indicating significant gene flow within each location, which is consistent with the invasive history of this species in France based on the literature. STRUCTURE analysis confirmed that all sampled populations originated from the introduction area of Mazamet, and Fig. 2 illustrates the role of invasive history consistent with the pattern of genetic variation. Low DW values in the most recently invaded and remote climatic zone, i.e. the Pyrenean mountains, reinforced the role of GD and invasion history in the present genetic structure of French populations. The results suggest that during Pyrenean mountain population expansion, where colonization and gene flow are potentially hampered by natural barriers [7], rare alleles were lost; however, genetic diversity (H) was maintained. Despite differences in spatial resolution, the support we found for GD is consistent with genetic variation in populations at the continental scale. Lachmuth et al. [33] reported that genetic variation increases with *S. inaequidens* population age, and several isolated populations are bottlenecked across Europe. Our AFLP analysis also showed evidence for high within-population genetic diversity, which is expected in an outcrossing tetraploid species.

It has to be noted that if the genetic differentiation observed is mainly driven by genetic drift and colonization history, it is not expected to explain invasion success in contrasted climatic environments.

### Environmental maternal effects: interplay between seed mass and climate

Seed mass was an important source of phenotypic variation in the Pyrenean garden. In contrast, EME were considerably lower under favorable spring climatic conditions in the Mediterranean garden. This result, when evaluated with a previous Belgium study performed in a rather mild climate [22], strongly suggests that EME only operate under harsh spring climatic conditions. Our results therefore document how the climatic environmental conditions influenced EME expression and, consequently, offspring performance.

Previous glasshouse experiments suggest that experimental conditions influence EME expression, e.g. competition and light [58], [59] and, in the field, the light regime has been reported to affect the importance of seed mass on progeny traits [60], [61]. In the present study, spring climatic conditions imposed a great influence on EME expression in the experimental gardens. Large-scale germination occurred under the mild Mediterranean climate, which lowered the potential influence of seed mass on phenotypic traits. However, when harsher climatic conditions resulted in prolonged germination, seed mass markedly affected germination timing and fitness-related traits. Heavier, earlier-germinating seeds produced larger, longer-lived individuals, which eventually produced an increased number of flower heads throughout the growing season. EME are generally reported to affect only plant juvenile stages [20]. However, our results indicate that EME, at least in a particular environment, can affect fitness over an entire season.

Parental effects are unlikely to improve offspring fitness unless the environment is predictable relative to the maternal environment [60]. In *S. inaequidens*, climate may represent a particularly predictable parameter compared to known dispersal distances [36]. A larger seed mass may be an adaptive feature under harsher climatic conditions, crucial for the differential success of individuals. Altitudinal variation in seed mass was demonstrated in this study (cf. *field seed mass variation*). Monty and Mahy [27] suggested that response type is a form of PP. Taken together, this indicates that this species relies on adaptive trans-generational plasticity [62] to achieve successful development in mountain ecosystems.

### Conclusions

The results of this study indicate that *S. inaequidens*, one of the most rapid plant invaders in Europe, relies on concomitant and not mutually exclusive mechanisms for successful invasion of distinct climates, even at a regional scale. Consistent with recent advances in invasive plant evolution research [12], [57], our results indicate that GD and invasion history play a role in shaping the genetic structure of the species. The observed levels of PP and the climate-dependent importance of EME suggest a high potential for future migration to climatic zones not yet exploited. In addition, we found some support for adaptive EME, illustrating possible interactions between sources of phenotypic variation. This indicates that the independent study of evolutionary mechanisms can lead to erroneous interpretations of their actual importance. For example, eliminating EME by growing plants for one generation in a controlled environment [20] can lead to an overestimation of the role of LA. Our results also suggest that the “*local adaptation* vs. *phenotypic plasticity*” question, frequently addressed in the literature on invasive plant trait variation in the invaded range [17], [18], [19], is not totally sufficient to fully understand what shapes phenotypic variation and genetic architecture. We argue that the question could be widened by including EME, GD and invasion history.

As a result, if understanding the mechanisms responsible for phenotypic variation in relation to an environmental factor is the goal, not only is a reciprocal experiment preferred to single gardens, but sowing is also preferable to transplantation. In addition, garden experiments coupled with genetic analyses provide a more robust analysis. This represents a research opportunity for ecologists to understand how invasive plant populations face gradual environmental changes during colonization.

## Supporting Information

Table S1 Geographic characteristics of *Senecio inaequidens* populations sampled in each climatic zones, along with the number of plants sampled for the AFLP analysis (n), the genetic variation (H), and the rarity index (DW).
(DOC)

Table S2 Monthly climatic data during the experiment (March–December 2008) in both experimental gardens, based on climatic stations near the experimental sites.
(DOC)
