# Supplementary material for: Rapid Plant Invasion in Distinct Climates Involves Different Sources of Phenotypic Variation
Source: PLoS One. 2013 Jan 30;8(1):e55627. doi: 10.1371/journal.pone.0055627 (PMC3559535; doi:10.1371/journal.pone.0055627)
Supplement: Table S1 — Geographic characteristics of Senecio inaequidens populations sampled in each climatic zones, along with the number of plants sampled for the AFLP analysis (n), the genetic variation (H), and the rarity index (DW). (DOC) [file pone.0055627.s001.doc]

| Climatic zone | Location | Altitude (m) | Latitude N | Longitude E | n | DW | H (SE) |
| --- | --- | --- | --- | --- | --- | --- | --- |
| Mediterranean zone |  |  |  |  |  |  | 0.282 (0.015) |
|  | Narbonne | 5 | 43°11' | 3°02' | 20 | 13.8 | 0.290 (0.015) |
|  | Narbonne-Plage | 2 | 43°10' | 3°11' | 20 | 16.2 | 0.300 (0.015) |
|  | Armissan | 135 | 43°10' | 3°06' | 20 | 13.4 | 0.277 (0.016) |
| Introduction zone |  |  |  |  |  |  | 0.284 (0.015) |
|  | Mazamet | 315 | 43°28' | 2°22' | 20 | 14.7 | 0.290 (0.015) |
|  | Castaunouze | 370 | 43°29' | 2°23' | 20 | 14.2 | 0.267(0.015) |
|  | Hautpoul | 415 | 43°28' | 2°23' | 20 | 16 | 0.303 (0.015) |
| Pyrenean zone |  |  |  |  |  |  | 0.298 (0.016) |
|  | La Llagone | 1695 | 42°32' | 2°08' | 20 | 12.9 | 0.316 (0.017) |
|  | Egat | 1635 | 42°30' | 2°01' | 20 | 13.2 | 0.293 (0.016) |
|  | Enveitg | 1416 | 42°28' | 1°54' | 20 | 12.5 | 0.289 (0.016) |
