# Supplementary material for: Rapid Plant Invasion in Distinct Climates Involves Different Sources of Phenotypic Variation
Source: PLoS One. 2013 Jan 30;8(1):e55627. doi: 10.1371/journal.pone.0055627 (PMC3559535; doi:10.1371/journal.pone.0055627)
Supplement: Table S2 — Monthly climatic data during the experiment (March–December 2008) in both experimental gardens, based on climatic stations near the experimental sites. (DOC) [file pone.0055627.s002.doc]

|  | Mediterranean garden | | | | | Pyrenean garden | | | | | |
| --- | --- | --- | --- | --- | --- | --- | --- | --- | --- | --- | --- |
|  | Mean minimal temperature (°C) | Mean maximal temperature (°C) | Minimum temperature (°C) | Maximal temperature (°C) | Cumulated rainfall (mm) | Mean minimal temperature (°C) | | Mean maximal temperature (°C) | Minimum temperature (°C) | Maximal temperature (°C) | Cumulated rainfall (mm) |
| March | 4.8 | 15.7 | -2 | 22.5 | 19.1 |  | -0.7 | 7.6 | -7 | 7.6 | 84.6 |
| April | 7 | 18.2 | 2 | 26.5 | 63.5 |  | 2.1 | 13.2 | -4.5 | 13.2 | 71.6 |
| May | 12.4 | 22 | 6 | 27 | 136.6 |  | 5.4 | 14.2 | 1 | 14.2 | 163.5 |
| June | 15.7 | 26.7 | 10.5 | 33.5 | 70.6 |  | 8.3 | 18.9 | 4 | 18.9 | 46.9 |
| July | 17.4 | 29.4 | 14 | 33 | 24.9 |  | 8.6 | 22.7 | 3.5 | 22.7 | 45.1 |
| August | 16.7 | 29.5 | 12.5 | 36 | 5 |  | 9.6 | 23.6 | 5 | 23.6 | 40.3 |
| September | 12.4 | 25.1 | 6.5 | 32 | 18.3 |  | 6.2 | 19.1 | 0 | 19.1 | 42.6 |
| October | 9.8 | 20.3 | 2 | 27.5 | 115.2 |  | 3.5 | 14.3 | -6 | 14.3 | 63.3 |
| November | 5.1 | 14.9 | -2.5 | 20.5 | 139.9 |  | -0.7 | 6.4 | -10 | 6.4 | 86.7 |
| December | 1.7 | 10.8 | -5.5 | 17.5 | 180 |  | -2.2 | 5.3 | -8 | 5.3 | 24.8 |
